# Cystic Fibrosis: Systems Biology Analysis from Homozygous p.Phe508del Variant Patients' Samples Reveals Perturbations in Tissue-Specific Pathways

**DOI:** 10.1155/2021/5262000

**Published:** 2021-12-02

**Authors:** Joice de Faria Poloni, Thaiane Rispoli, Maria Lucia Rossetti, Cristiano Trindade, José Eduardo Vargas

**Affiliations:** ^1^Laboratório de Bioinformática Estrutural e Biologia Computacional, Instituto de Informática, Universidade Federal do Rio Grande do Sul, Porto Alegre, RS, Brazil; ^2^Laboratório de Bioinformática em Bioenergia (LBB), Embrapa Agroenergia Parque Estação Biológica, Brasília, DF, Brazil; ^3^Programa de Pós-Graduação em Biologia Celular e Molecular, Universidade Federal do Rio Grande do Sul, Porto Alegre, RS, Brazil; ^4^Centro de Desenvolvimento Científico e Tecnológico (CDCT), Centro Estadual de Vigilância em Saúde (CEVS) Secretaria da Saúde do Estado do Rio Grande do Sul (SES-RS), Porto Alegre, RS, Brazil; ^5^Programa de Pós-Graduação em Biologia Celular e Molecular Aplicada à Saúde, Universidade Luterana do Brasil, Porto Alegre, RS, Brazil; ^6^Facultad de Ciencias Básicas y Biomédicas, Universidad Simón Bolívar, Barranquilla, Colombia; ^7^Programa de Pós-graduação Ciências em Gastroenterologia e Hepatologia, Hospital de Clínicas de Porto Alegre, Porto Alegre, Brazil

## Abstract

Cystic fibrosis (CF) is an autosomal recessive disorder, caused by diverse genetic variants for the CF transmembrane conductance regulator (CFTR) protein. Among these, p.Phe508del is the most prevalent variant. The effects of this variant on the physiology of each tissue remains unknown. This study is aimed at predicting cell signaling pathways present in different tissues of fibrocystic patients, homozygous for p.Phe508del. The study involved analysis of two microarray datasets, E-GEOD-15568 and E-MTAB-360 corresponding to the rectal and bronchial epithelium, respectively, obtained from the ArrayExpress repository. Particularly, differentially expressed genes (DEGs) were predicted, protein-protein interaction (PPI) networks were designed, and centrality and functional interaction networks were analyzed. The study reported that p.Phe508del-mutated CFTR-allele in homozygous state influenced the whole gene expression in each tissue differently. Interestingly, gene ontology (GO) term enrichment analysis revealed that only “neutrophil activation” was shared between both tissues; however, nonshared DEGs were grouped into the same GO term. For further verification, functional interaction networks were generated, wherein no shared nodes were reported between these tissues. These results suggested that the p.Phe508del-mutated CFTR-allele in homozygous state promoted tissue-specific pathways in fibrocystic patients. The generated data might further assist in prediction diagnosis to define biomarkers or devising therapeutic strategies.

## 1. Introduction

Cystic fibrosis (CF) [OMIM (Online Mendelian Inheritance in Man: #219700)] is a monogenic disease, which is caused by the occurrence of more than 2,000 genetic variants for the protein CF transmembrane conductance regulator (CFTR) [1, 2]. Among these, p.Phe508del is known to be most prevalent CFTR variant. It is responsible for ~70% of CF cases across the globe (http://www.genet.sickkids.on.ca/andhttps://www.cftr2.org/). In particular, this variant involves a three bp deletion in exon 10 that results in the absence of amino acid phenylalanine at position 508 of the final protein (c.1521_1523delCTT, F508del or p.Phe508del) [3].

The CFTR protein is generally located at the apical membrane of polarized epithelial cells present in the respiratory tract, submucosal glands, gastrointestinal tract, exocrine pancreas, liver, sweat ducts, reproductive tract, and other tissues [4]. In particular, this protein functions as a chloride ion channel that controls the movement of ions (Cl^−^), water secretion, and absorption in the epithelial tissues. The channel activation is mediated via cycles of phosphorylation of the regulatory domain, ATP-binding to the nucleotide-binding domains, and ATP hydrolysis [5]. Thus, absence or dysfunction of CFTR results in an ionic imbalance, secretion of thick and dehydrated mucus, and fat malabsorption, which further lead to different CF phenotypes [6].

Recently, different CFTR variants were divided into seven classes on the basis of mechanisms that mediated the qualitative and quantitative variations in the expression of CFTR. Additionally, this classification system also considered the availability/applicability of precision medicine [7–10]. Although the morbidity and mortality related to CF are mostly associated to the respiratory system, the researchers have started to explore and understand the implications of CFTR variants on the entire gastrointestinal (GI) system. In fact, several studies have previously shown that inflammation exerts both local and systemic effects [9].

Even though a large number of CFTR variants have been described, very limited information is available regarding the pathway perturbations promoted by the classical and most prevalent CFTR variant, p.Phe508del, and its associated effects. This study is aimed at evaluating and analyzing the gene expression profiles for the bronchial and rectal tissues of CF subjects homozygous for p.Phe508del variant, using systems biology approaches. The findings of the study would assist in improving current understanding regarding the physiology of each tissue, which could further help in devising new and improved therapeutic strategies.

## 2. Materials and Methods

### 2.1. Protein-Protein Interaction (PPI) Network Design

To analyze cell signaling pathways for different tissues of CF patients, microarray datasets were collected from the ArrayExpress repository [11]. The inclusion criteria for the study considered gene expression datasets obtained from CF patients' samples homozygous for the p.Phe508del-mutated CFTR-allele. In particular, two datasets fulfilled these requirements, namely, E-GEOD-15568 and E-MTAB-360 that corresponded to the rectal [12] and bronchial epithelium [13], respectively. The rectal epithelium dataset was composed of 13 non-CF and 16 CF patients, whereas the bronchial epithelium dataset was composed of nine non-CF and 19 CF patients. The raw data sets were downloaded using ArrayExpress package and analyzed using the R package arrayQualityMetrics [14, 15]. The analysis for differentially expressed genes (DEGs) was performed using the R package limma, wherein ∣log2FC | >0.5 and adjusted *P* < 0.05 were used as cut-off values [16].

### 2.2. Protein-Protein Interaction (PPI) Network Design

The DEGs for each dataset were used as input to design the protein-protein interaction (PPI) network, using the metasearch engine STRING 10.5. For each tissue, an individual network was created [17]. For PPI network design, the parameters that were employed involved prediction methods enabled for “experiments,” “databases,” and “coexpression” and minimum confidence value of interactions to be 0.4, with no more than 20 interactors in the first shell and no more than five interactors in the second shell. According to the aforementioned protocol, the nonconnected nodes were repeatedly provided as an input until no more connections were found. Further, all subnetworks were merged in the software Cytoscape 3.7.2 [18, 19]. An additional network was created using CFTR as input, and the most relevant nodes were obtained from the centrality analysis (see Section 2.3), wherein the parameters involved predictions methods enabled for “experiments,” “databases,” and “coexpression” and minimum confidence value of interactions of 0.4, with no more than 50 interactors in the first shell and no interactors in the second shell. This network was grown to the saturation, and only interactions between the inputs were retained.

### 2.3. Centrality Analysis

To assess the topological relevance of the network, the centrality analysis was performed using the Cytoscape plugin CentiScape 2.2 [20, 21]. For centrality analysis, node degree was used to evaluate the node connectivity by calculating adjacent neighbors, betweenness was used to calculate the shortest paths connecting adjacent nodes that pass through each node, and eigenvector was utilized to measure the regulatory potential of a given node based on the relevance of its neighbors. In general, nodes showing above-average scores on the node degree analysis are named “Hub,” nodes showing above-average scores for betweenness analysis are denoted as “Bottleneck,” and those with above-average scores for eigenvector analysis are termed as “Switch.” Thus, nodes combining the three centralities above-average scores are termed as HBS, which denotes a node with a robust regulatory role and a strong influence on the network [21, 22]. Further, the identified HBS nodes were used as input in the Reactome FIViz app to construct a functional interaction network, with the aid of the information obtained from the Reactome database [23].

### 2.4. Gene Ontology Analysis

Gene Ontology (GO) analysis for DEGs was performed using topGO and clusterProfile, wherein both were performed on R platform [24]. GO results and gene expression data were integrated and plotted using GOplot [25]. GO analysis for DEGs was performed using the default settings, and the results showing FDR < 0.05 were considered to be relevant. Additionally, GO analysis for the PPI network was performed to verify the enriched biological processes related to the DEGs for HBSs (HBS-DEGs), using aforementioned packages, and FDR < 0.001 was applied as cut-off value.

Module discovery and GO analysis for HBS functional interaction network were performed using Reactome FIViz app [23]. The results were recovered by applying filter of FDR < 0.001.

## 3. Results and Discussion

**Figure 1:**
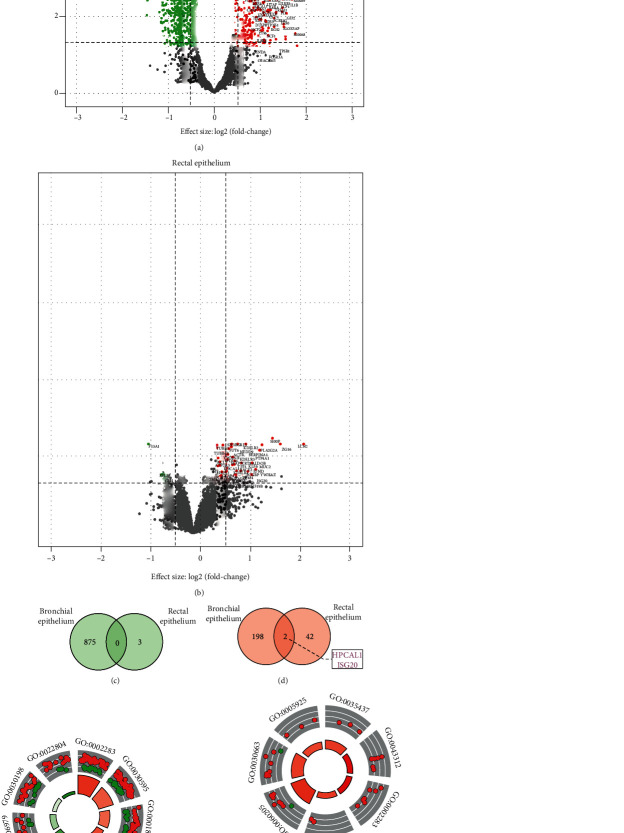
Summary for the differential expression analysis. Volcano plot for the distribution of over and underexpressed genes in the (a) bronchial epithelium and (b) rectal epithelium. Venn diagram for the underexpressed and overexpressed genes for the (c) bronchial epithelium and (d) rectal epithelium. Visualization of the results for the DEGs enrichment analysis (cut-off FDR < 0.05) obtained from the (e) bronchial epithelium and (f) rectal epithelium. In the inner ring, bar height indicates GO's significance (−log10 adjusted *P* value), while the color corresponds to the *z*-score, which is measured according to the gradient of color bar. The outer ring represents the log2FC dispersion value for the DEGs associated with each GO. Green: underexpressed genes; red: overexpressed genes.

The comparison of gene expression profiles for the rectal and bronchial epithelium for CF patients, homozygous for the p.Phe508del variant, was compared with non-CF patients. For bronchial epithelium, a total of 1075 DEGs were identified, wherein 200 genes were found to be overexpressed, and 875 genes were underexpressed. The results for these DEGs are depicted in the volcano plot shown in Figure 1(a). In case of the rectal epithelium, 47 DEGs were identified, wherein 44 were overexpressed, while three genes were underexpressed genes (Figure 1(b)).

The discrepant DEGs identified between the rectal and bronchial epithelium showed that CF condition did not promote the expression of similar genes in these tissues. To verify the absence of any similarity between these tissues, DEGs were overlapped between both epithelia (Figures 1(c) and 1(d)).

Interestingly, the results for the analysis showed that only two of the overexpressed genes were shared by both tissues, namely, interferon-stimulated exonuclease gene 20 (ISG20) and hippocalcin-like protein 1 (HPCAL1). With regard to CF, no previous data have directly reported any involvement of ISG20 pathways in CF patients. However, ISG20 could be relevant for CF pathogenesis, wherein broad antiviral properties could have attributed to its expression. In particular, this gene codes for a nuclear 3′-5′ exonuclease, which degrades viral RNAs as part of the interferon- (IFN-) regulated antiviral response [26–28].

In fibrocystic patients, viral respiratory infections are known to promote deterioration of lung functions [29, 30], which further results in severe respiratory morbidity in children [31]. Additionally, the presence of exacerbated symptoms in the bronchial epithelium of CF patients were found to be associated with the presence of viruses, including respiratory syncytial virus [32], influenza types A and B [32, 33], parainfluenza [34], and human rhinovirus [35]. The results for *in* vitro assessment using human alveolar macrophages showed that H3N2 also promoted the expression of ISG20 [36]. However, it is important to note that the expression of ISG20 was not restricted to the lungs. In fact, this gene was previously shown to be expressed at high levels in the peripheral blood leukocytes, lymphoid tissues (spleen or thymus), and the colon [37]. Additionally, this gene was also expressed in diverse human-tumor types [38, 39].

Hippocalcin-like protein 1 (HPCAL1) is a neuronal calcium sensor (NCS) protein that has been majorly described in the brain [40]. Although its expression has also been reported in different cellular and tumoral models, including the lungs [41], the biological function of HPCAL1 in CF pathogenesis remains unknown.

Importantly, these two DEGs represent only 1% (2/200) of the overexpressed genes for the bronchial epithelium and 4.5% (2/44) for the rectal epithelium of CF samples. In terms of the total number of DEGs, this percentage further reduced to 0.18% (2/1075) and 4.2% (2/47) for the bronchial and rectal epithelia, respectively. This data further supports that the presence of p.Phe508del-mutated CFTR-allele in homozygous state influences the whole gene expression differently in each analyzed tissue.

In contrast to previously established idea, genes can belong to multiple groups at the same level. Thus, a DEG list might be statistically overrepresented in a GO category. To establish the same, GO term enrichment analysis was performed to predict the shared GO terms between the bronchial and rectal epithelia of CF patients (refer to the methods section for more details).

As shown in Figure 1(e), the biological processes for DEGs were found to be associated with immunological responses, namely, inflammatory response (GO:0056729), leukocyte chemotaxis (GO:0030595), and cytokine production (GO:0001819), in case of the bronchial epithelium. In comparison to this, GO terms for the rectal tissue were majorly related to the intracellular transport, involving cytoplasmatic vesicle lumen (GO:0060205), COPI-coated vesicle membrane (GO:0030663), focal adhesion (GO:0005925), and maintenance of protein localization in the endoplasmic reticulum (GO:0035437) and organelles (GO:0072595) (Figure 1(f)). According to this data, the majority of cellular functions were unrelated between these tissues. However, neutrophil activation (GO:0002283) appeared to be shared by the two tissues. Several previous studies have shown that infants with CF exhibited peribronchial neutrophilic infiltration prior to an initial infection [42–44]. In toddlers and older children, the inflammatory response is known to increase gradually, which further stimulates changes in mucus viscosity and affects bronchiolar function [45, 46]. Moreover in older patient, high levels of airway cytokines (e.g., IL−8) were also reported in addition to the neutrophil influx, which aggravated the lung complications [42, 47]. These inflammatory lung conditions facilitate growth of opportunistic and chronic infections at different stages of the lung disease [48].

In the gastrointestinal tract, neutrophil activation and NETosis have also been reported in the colon mucosa of ulcerative colitis in fibrocystic patients [49]. In general, NETosis refers to the process of cell death that is related to the formation of neutrophil extracellular traps (NETs). The formation of NETs has been previously described in the airways of patients with CF, wherein tissue contamination by different microorganisms can lead to an exacerbated immune response [50]. However, this excessive formation of NETs results in the worsening of patient's condition due to tissue damage caused by the components of NETs [50].

**Figure 2:**
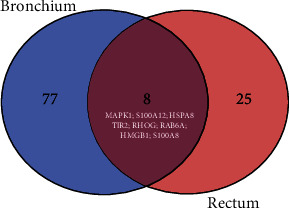
Venn diagram to compare the predicted genes for GO:0002283 between the rectal and bronchial tissues.

As shown in Figure 2, nonshared DEGs were grouped into the same GO term (GO:0002283) between the bronchial and rectal tissues. A Venn diagram was prepared to compare all the predicted genes for each tissue into this GO term. The results showed that eight genes were shared between these tissues; however, these genes did not show differential expression. This data further suggested that each tissue involved different signaling pathways that contributed to the induction of a similar neutrophil phenotype.

**Figure 3:**
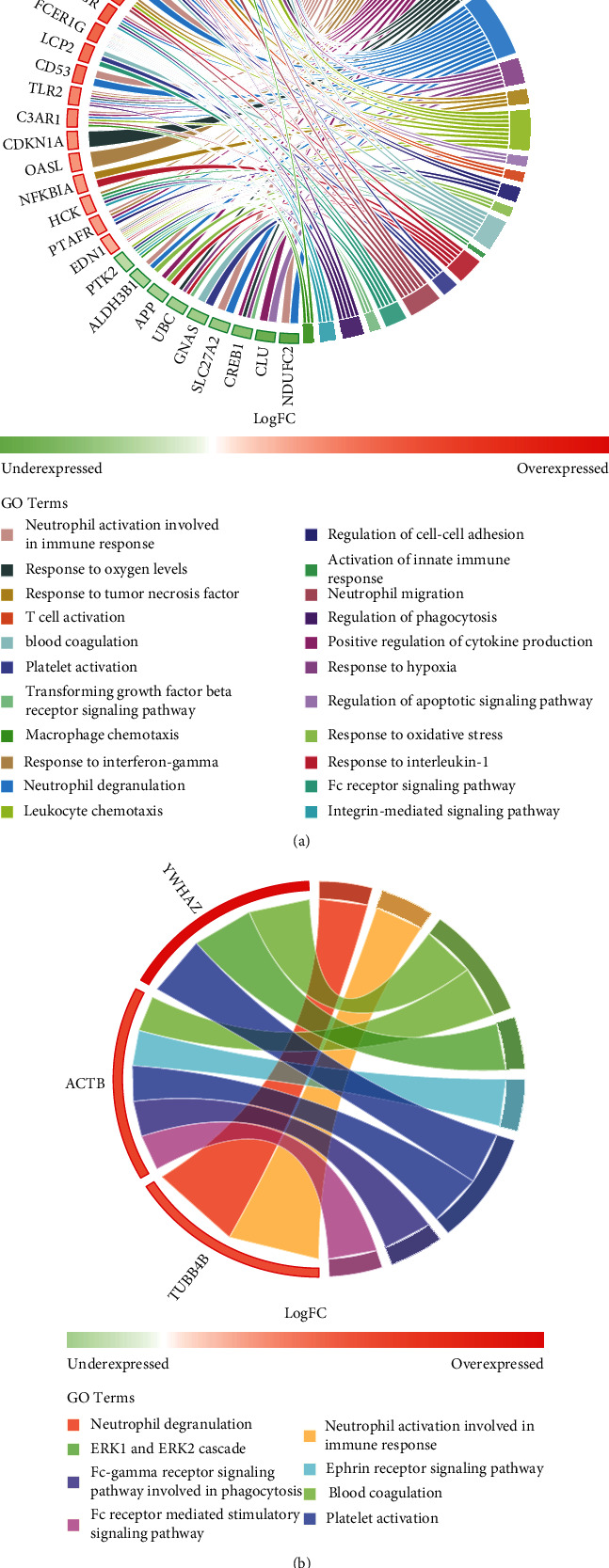
HBS-DEGs were selected from the pool of significant GO terms related to the (a) entire bronchial epithelium and (b) rectal epithelium networks (cut-off FDR < 0.001). These are depicted using the ribbons that link each GO to bronchial and rectal HBS-DEGs, respectively.

To verify this hypothesis, an interatomic approach was adopted and performed between all DEGs. In general, a network strategy allows application of topological analysis to decipher pathways involved in each tissue. To achieve a systemic perspective for CF, PPI networks were first constructed for the bronchial and rectal tissues (for details refer to the methods section). Consequently, a bronchial PPI network comprising of 1,143 nodes and 8,091 edges was generated (Supplementary Figure 1). In comparison to this, the rectal PPI network was composed of 342 nodes and 2,178 edges (Supplementary Figure 2). Following this, topological centrality analyses were conducted for both networks to define HBSs for each tissue (Supplementary Figure 3a and 3b). In the bronchial PPI network, 175 HBSs were predicted, wherein 30 nodes corresponded to DEGs associated with one or more interrelated ontologies (Figure 3(a)). A similar observation was reported in case of the rectal PPI network, wherein a total of 45 HBSs were predicted. Among these, three nodes, namely, ACTB, TUBB4B, and YWHAZ, showed differential expression. Interestingly, these nodes were also associated with multiple GO for the rectal PPI network (Figure 3(b)).

Thus, these results suggested that no HBSs were shared between the bronchial and rectal PPI networks, which further confirmed that tissue-specific pathways were associated with the homozygous p.Phe508del variant. Additionally, the terms predicted from GO analysis of DEG (Figures 1(e) and 1(f)) and HBS networks were found to be similar (Figures 3(a) and 3(b)) and shared a GO term that was related to neutrophil activation.

This data highlighted that these HBSs represented relevant biological processes for each tissue; however, no possible regulatory relationships were identified between them at the pathway level. Integrated pathway analyses were employed to capture such tissue-specific signatures.

**Figure 4:**
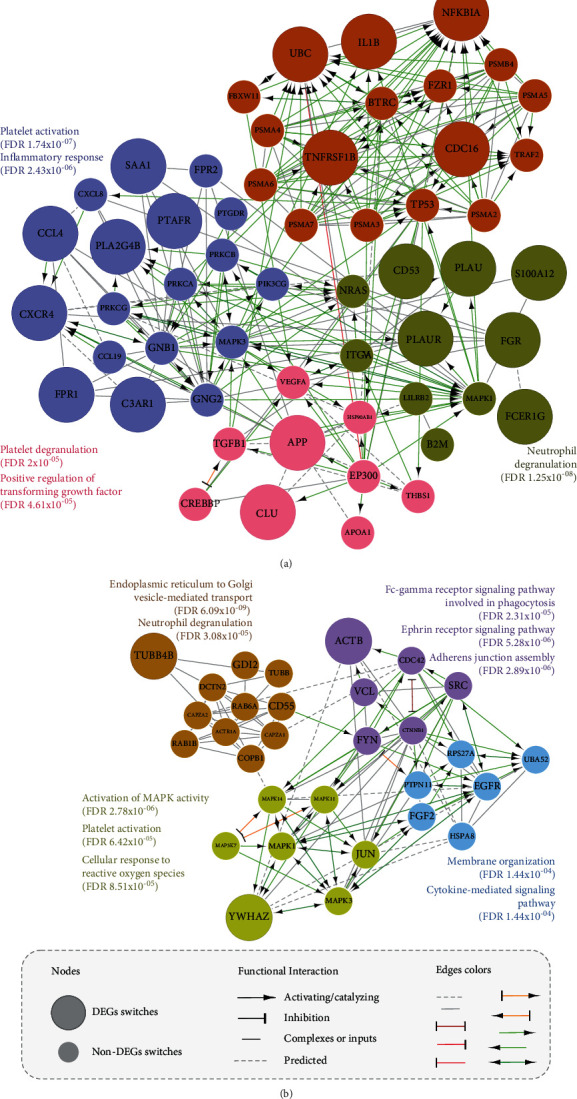
Pathway-based networks with the most significant GOs (cut-off FDR < 0.001) for the HBSs of the (a) bronchial epithelium and (b) rectal epithelium.

To gain further insights into the potential relationship between the bronchial and rectal epithelia and tissue-specific response at the pathway level, the HBSs were used to construct functional interaction networks (Figure 4). For bronchial tissue, 20 HBS-DEGs and 35 HBS-non-DEGS were predicted to interconnect the pathways that were majorly composed of activators (Figure 4(a)). Similarly, the rectal epithelium pathways involved only three HBS-DEGs and 27 HBS-non-DEGs (Figure 4(b)). Interestingly, one unique node, MAPK3, a non-DEG, was shared between the pathway-based networks for the two tissues. This observation further confirmed the induction of independent cell signaling processes in each tissue by the homozygous p.Phe508del variant. Moreover, it was possible to discriminate the GO term named neutrophil degranulation at pathway level, where FDR = 1.25 × 10^−08^ and FDR = 3.08 × 10^−05^ were used for the bronchial and rectal tissues, respectively. Degranulation from neutrophils has been previously implicated as a significant causative factor in pulmonary disorders, which is dependent of neutrophil activation [51]. Post the analysis of this GO term, no shared HBSs were found between two tissues. In case of the bronchial tissue, CD53, PLAUR, PLAU, S100A12, FGR, and FCER1G were predicted, whereas only TUBB4B was predicted for the rectum. This result verified the initial hypothesis that different tissue-specific pathways could possibly promote a similar neutrophil phenotype.

Interestingly, the other GO term that emerged to be common between the analyzed tissues, obtained from Reactome database, was platelet activation, wherein FDR = 2 × 10^−05^ and FDR = 6.43 × 10^−05^ were used for the bronchial and rectal tissue, respectively (Figure 4). Platelets are known to be essential players in the development of inflammatory response, which is attributed to their interaction with leukocytes and their role in the secretion of proinflammatory mediators and migratory behavior cells [52]. In CF patients, increased levels of circulating leukocyte-platelet aggregates have been reported [53–55]. For this GO term, SAA1, CCL4, PTAFR, PLA2G4B, CXCR4, FPR1, and C3AR1 HBSs were predicted for the bronchial epithelium. However, only YWHAZ HBS was found to be associated with this term in case of the rectal tissue. This observation further reinforced the idea that different tissue-specific pathways could induce a similar cellular response.

In the last few year, significant additions have been made to the list of key genes involved in CF [56–61], and the identification of essential disease-related pathways remains a priority, particularly for the development of new treatment strategies. For the first time, the present study defined tissue-specific pathways stratified for the fibrocystic genotype, .p.Phe508del-mutated CFTR-allele in homozygous state. Consequently, the use of in silico strategy in the present study resulted in the identification of putative tissue-specific biomarkers and unraveled their association at the systemic level. To verify this, previous studies were reviewed to find a relationship between each HBS-DEG and CF, based on gene expression data (Table 1).

**Figure 5:**
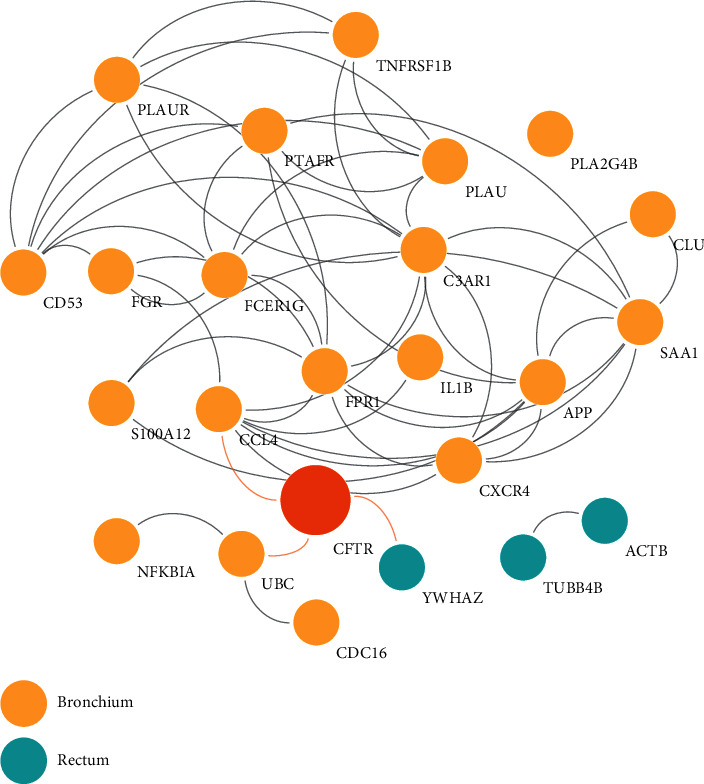
PPI network generated between CFTR and HBSs found in the bronchial and rectal epithelial networks.

As shown in Table 1, review of gene expression data reported in previous studies revealed that APP, FPR1, PTFAR1, TNFRSF1B, UBC, and YWHAZ were not reported for CF. An additional network was generated to decipher its tentative functions and predict the interaction between all HBS-DEGs included in Table 1 and CFTR (Figure 5). Interestingly, TNFRSF1B, UBC, and YWHAZ directly modulated this receptor. However, the involvement of these HBS nodes in CF pathogenesis still remains unknown. These three nodes might affect the structural stability of the CF receptor.

TNFRSF1B generally encodes for a high-affinity receptor for tumor necrosis factor (TNF) *α*. In terms of CF, this protein might influence the trafficking of F508del-CFTR through the Golgi apparatus by regulating the levels of TNF-*α* in bronchi. In a previous *in vitro* study, F508del-CFTR-transfected HeLa cells and human bronchial cells expressing F508del-CFTR in primary culture were exposed to TNF-*α* (0.5–50 ng/ml) for 10 min [87]. This treatment promoted the maturation of F508del-CFTR via Golgi vesicular transport and induced CFTR chloride currents. According to this evidence, TNF-*α* and TNFRSF1B balance could be essential for the maturation of CFTR, but this hypothesis still needs to be experimentally tested. At the genomic level, TNFRSF1B polymorphisms have been shown to be associated with severe pulmonary phenotype in CF [88].

In comparison to this, the polyubiquitin gene ubiquitin C (UBC) is considered to be a stress-protective gene, which is upregulated under various stressful conditions, probably as a consequence of the increased demand for ubiquitin for the removal of toxic misfolded proteins [89]. In the context of CF, misfolding of the CFTR proteins has been described for CF patients in several previous studies. In particular, F508del-CFTR mutation is known to be a major cause of 70% of CF cases [90]. In the present study, UBC was downregulated, which further suggested that the degradation of machine-mediated polyubiquitin is also affected in CF patients. Some previous studies have addressed this point, wherein failure in proteasome degradation maintained aberrantly folded CFTR proteins [91, 92].

YWHAZ, a member of 14-3-3 proteins, is a conserved regulatory protein that maintains multiple types of signals via binding to several partner proteins. The binding of 14-3-3 proteins can lead to conformational changes in their partners, masking specific sequences or structural features in the partner proteins that promote the formation of complex [93]. No previous data has reported YWHAZ interaction with CFTR. However, this family of proteins is known to affect a consensus sequence, RXXpS/TXP [94]. It might enforce conformational changes in the binding partner around the phosphorylated docking sites. Future studies are required to explore these conformational changes and elucidate their association with CF phenotypes.

The present study was associated with certain limitations. In this study, gene transcription analysis was performed for a total of 35 CF patients and 22 healthy controls. Complementary studies should be carried out in independent cohorts with a higher number of tissues. However, this limitation, a low size sample, is recurrent and has been reported in other exploratory works also, which involved different criteria (inclusion/exclusion) for screening of potential markers or key pathways in CF [58, 71, 95, 96]. Additionally, it is crucial to verify whether the p.Phe508del variant in the homozygous state promotes pathways perturbations in several tissues or exclusively in the bronchial and rectal epithelia. Moreover, patients should be followed longitudinally to correlate the genetic expression of the predicted HBSs with the clinical outcomes in the future.

Finally, it is essential to note that choosing an accurate genotyping method for diagnosis and a priori knowledge of population's genetic variations is critical to design putative biomarkers or treatments. For example, the frequency of homozygous p.Phe508del variant changes according to ethnicity [97]. Thus, considering all these points could increase the relevance of our findings.

## 4. Conclusions

The present study assessed the genetic expression profiles for the bronchial and rectal samples obtained from CF patients homozygous for the p.Phe508del-mutated CFTR-allele and identified certain tissue-specific pathways. The integration of the results of GO analyses for DEGs and network strategies with topological analysis allowed the identification of HBSs for each tissue. Interestingly, each tissue possessed its unique HBSs that were involved in different cell signaling pathways, which promoted a similar cellular phenotype/response. The integrative approach utilized in this study offered comprehensive insights into the molecular networks for the bronchial and rectal epithelia and the underlying regulators involved in CF. The findings of the study might further aid in the development of tissue-specific therapeutics, based on genotypic analysis.

## Figures and Tables

**Table 1:** Literature review and data collection for the predicted HBS-DEGs in the context of CF pathogenesis. Only those studies were considered that explored gene expression data.

Symbol	Gene name	Network	Expression	CF-related	Tissue
APP	Amyloid beta precursor protein	Bronchial	Down	—	—
C3AR1	Complement C3a receptor 1	Bronchial	Up	Yes	Lung [62]
CCL4	C–C motif chemokine ligand 4	Bronchial	Up	Yes	Lung [63], Tear fluid [64]
CD53	CD53 molecule	Bronchial	Up	Yes	Nasal (airway inflammation) [65], Lung [66]
CDC16	Cell division cycle 16	Bronchial	Down	Yes	Blood neutrophils [67]
CLU	Clusterin	Bronchial	Down	Yes	Lung (airway secretions) [68]
CXCR4	C–X–C motif chemokine receptor 4	Bronchial	Up	Yes	Lung [69]
FCER1G	Fc fragment of IgE receptor Ig	Bronchial	Up	Yes	Lung [66, 70], Pancreas [71], Intestine [72]
FGR	FGR protooncogene, Src family tyrosine kinase	Bronchial	Up	Yes	Kidney [73]
FPR1	Formyl peptide receptor 1	Bronchial	Up	—	—
IL1B	Interleukin 1 beta	Bronchial	Up	Yes	Airway mucopurulent secretions [74, 75]
NFKBIA	NFKB inhibitor alpha	Bronchial	Up	Yes	Bronchial gland cells [76], Lung [77]
PLA2G4B	Phospholipase A2 group IVB	Bronchial	Down	Yes	Lung [78]
PLAU	Plasminogen activator, urokinase	Bronchial	Up	Yes	Airway epithelia [79]
PLAUR	Plasminogen activator, urokinase receptor	Bronchial	Up	Yes	Airway epithelia [79]
PTAFR	Platelet activating factor receptor	Bronchial	Up	—	—
S100A12	S100 calcium-binding protein A12	Bronchial	Up	Yes	Lung sputum [80–82]
SAA1	Serum amyloid A1	Bronchial	Up	Yes	Lung fibroblasts [83], Lung [62, 78], Blood [84]
TNFRSF1B	TNF receptor superfamily member 1B	Bronchial	Up	—	—
UBC	Ubiquitin C	Bronchial	Down	—	—
ACTB	Actin beta	Rectal	Up	Yes	Lung [13, 85]
TUBB4B	Tubulin beta 4B class IVb	Rectal	Up	Yes	Airway epithelia [86], ciliated cells [86]
YWHAZ	Tyrosine 3-monooxygenase/tryptophan 5-monooxygenase activation protein zeta	Rectal	Up	—	—

## Data Availability

Previously reported gene expression data were used to support this study and are available at doi:10.1038/ejhg.2013.209 and doi:10.1016/j.ygeno.2011.06.008. These prior studies (and datasets) are cited at relevant places within the text as references [11]; [12].

## Supplementary Materials

Supplementary Materials Supplementary Figure 1: bronchial PPI network comprising 1,143 nodes and 8,091 edges. Colored nodes (green to underexpressed genes; red to overexpressed genes) denote differential expression, and diamond shape indicates HBS nodes. Supplementary Figure 2: rectal PPI network comprising 342 nodes and 2,178 edges. Colored nodes denote log2FC of DEGS (green to underexpressed genes; red to overexpressed genes), and diamond shape indicates HBS nodes. Supplementary Figure 3: (a, b) visualization of the subnetworks from the HBS nodes of bronchial epithelium and rectal epithelium networks, respectively. Colored nodes indicate the log2FC of DEGs (green to underexpressed genes; red to overexpressed genes).
