# Supplementary material for: Cystic Fibrosis: Systems Biology Analysis from Homozygous p.Phe508del Variant Patients' Samples Reveals Perturbations in Tissue-Specific Pathways
Source: Biomed Res Int. 2021 Dec 2;2021:5262000. doi: 10.1155/2021/5262000 (PMC8660202; doi:10.1155/2021/5262000)
Supplement: Supplementary Materials — Supplementary Figure 1: bronchial PPI network comprising 1,143 nodes and 8,091 edges. Colored nodes (green to underexpressed genes; red to overexpressed genes) denote differential expression, and diamond shape indicates HBS nodes. Supplementary Figure 2: rectal PPI network comprising 342 nodes and 2,178 edges. Colored nodes denote log2FC of DEGS (green to underexpressed genes; red to overexpressed genes), and diamond shape indicates HBS nodes. Supplementary Figure 3: (a, b) visualization of the subnetworks from the HBS nodes of bronchial epithelium and rectal epithelium networks, respectively. Colored nodes indicate the log2FC of DEGs (green to underexpressed genes; red to overexpressed genes). [file 5262000.f1.docx]

***Supplementary Figures***

***
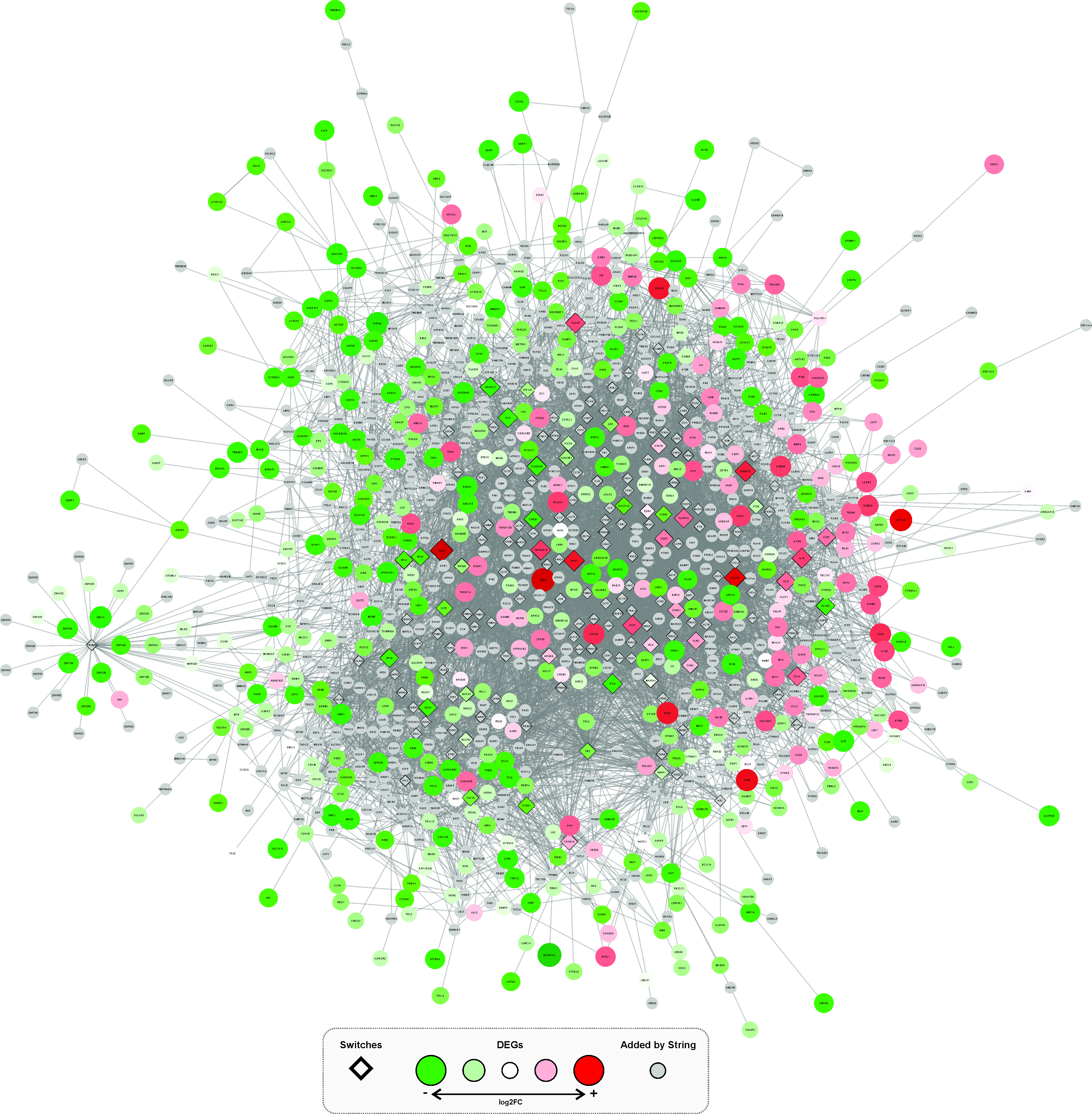
***

**Supplementary Figure 1.** Bronchial PPI network comprising 1,143 nodes and 8,091 edges. Colored nodes (green to underexpressed genes; red to overexpressed genes) denote differential expression and diamond shape indicates HBS nodes.

**
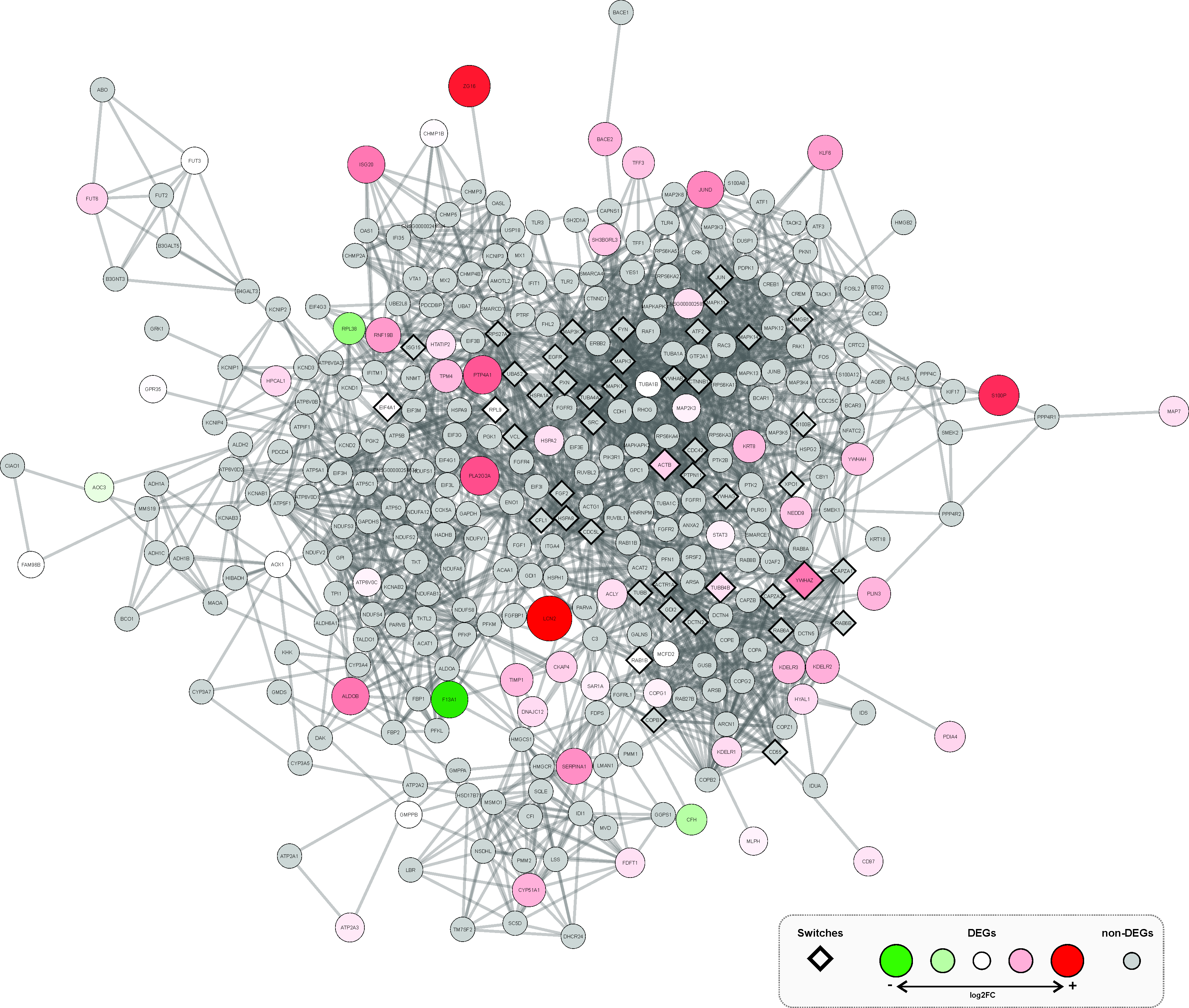
**

**Supplementary Figure 2.** Rectal PPI network comprising 342 nodes and 2,178 edges. Colored nodes denote log2FC of DEGS (green to underexpressed genes; red to overexpressed genes) and diamond shape indicates HBS nodes.

**
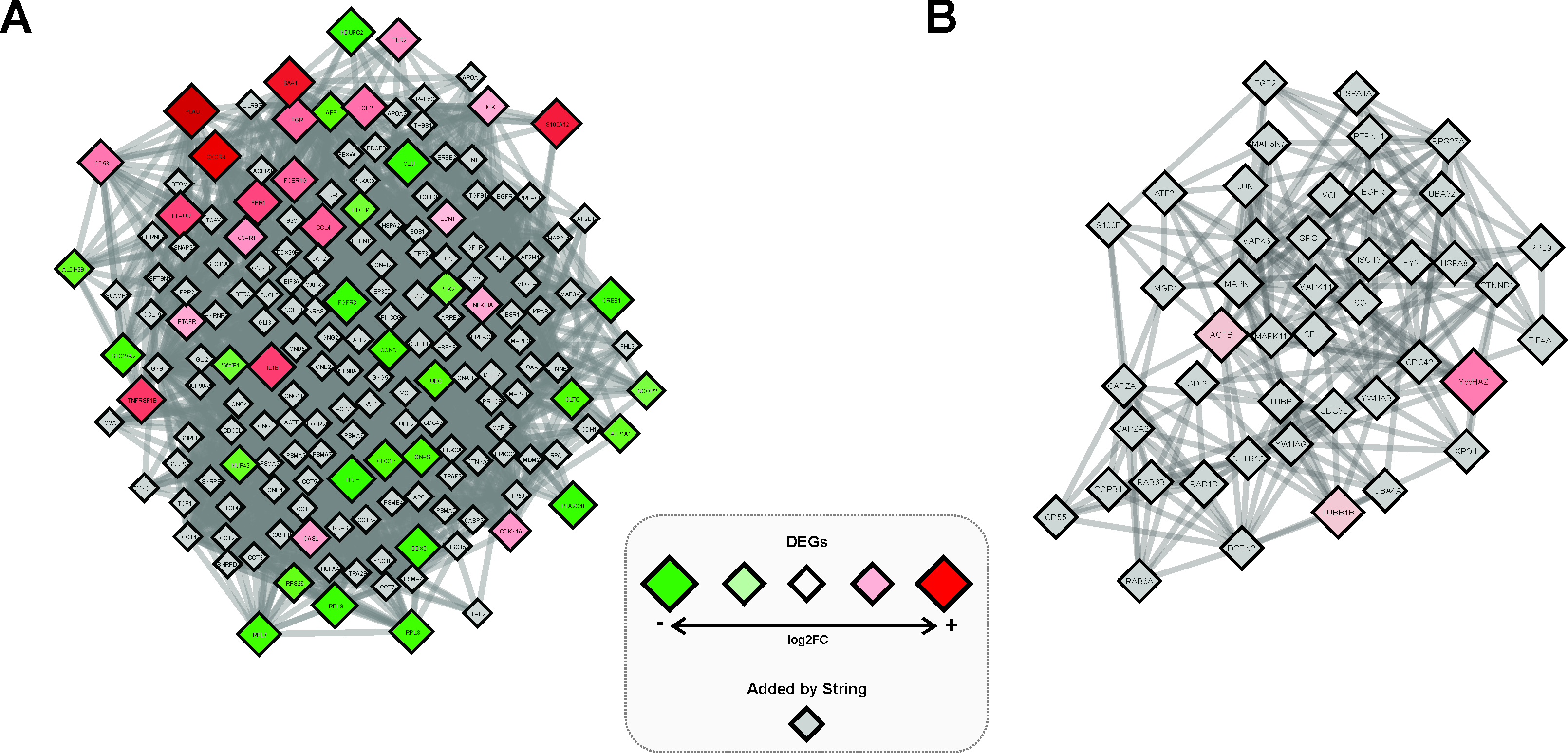
**

**Supplementary Figure 3. A-B)** Visualization of the subnetworks from the HBS nodes of Bronquial epithelium and Rectal epithelium networks, respectively. Colored nodes indicate the log2FC of DEGs (green to underexpressed genes; red to overexpressed genes).
